# A Modified Two-Temperature Calibration Method and Facility for Emissivity Measurement

**DOI:** 10.3390/ma18143392

**Published:** 2025-07-19

**Authors:** Shufang He, Shuai Li, Caihong Dai, Jinyuan Liu, Yanfei Wang, Ruoduan Sun, Guojin Feng, Jinghui Wang

**Affiliations:** 1 Division of Optical Metrology, National Institute of Metrology, Beijing 100029, Chinadaicaihong@nim.ac.cn (C.D.); sunrd@nim.ac.cn (R.S.);; 2 Academy of Artificial Intelligence, Beijing Institute of Petrochemical Technology, Beijing 102627, China; 3 Division of Thermophysics Metrology, National Institute of Metrology, Beijing 100029, China; wangjh@nim.ac.cn

**Keywords:** infrared radiation of materials, emissivity measurement, modified two-temperature calibration method, background radiation, direct method, indirect method

## Abstract

Measuring the emissivity of an infrared radiant sample with high accuracy is important. Previous studies reported on the multi- or two-temperature calibration methods, which used a reference blackbody (or blackbodies) to eliminate the background radiation, and assumed that the background radiation was independent of temperature. However, in practical measurements, this assumption does not hold. To solve the above problems, this study proposes a modified two-temperature calibration method and facility. The two temperature points are set in a certain small interval based on the proposed calculation method; based on the indication of the approximation that the emissivities of the sample and the background radiations remain the same at these two temperatures, the emissivities can be calculated with measurement signals at these two temperatures, and a reference blackbody is not needed. An experimental facility was built up and three samples with emissivities around 0.100, 0.500, and 0.900 were measured in (8~14) μm. The relative expanded uncertainties were 9.6%, 4.0%, and 1.5% at 60 °C, respectively, and 8.8%, 5.8%, and 1.2% at 85 °C (*k* = 2), respectively. The experimental results showed consistency with the results obtained using other methods, indicating the effectiveness of the developed method. The developed method might be suitable for samples whose emissivities are temperature insensitive.

## 1. Introduction

Emissivity means the ratio of the radiant exitance of an infrared radiant sample to that of a Planck radiator at the same temperature, and it is also equivalent to the ratio of their radiance [1]. To measure an emissivity value, there are usually direct and indirect methods. In the direct method, based on the definition of emissivity, a blackbody radiant source with emissivity close to one is used as a Planck radiator. By setting both the infrared radiant sample and the blackbody to the same temperature and measuring their radiance, the emissivity of the sample can be calculated accordingly [2,3,4]. While in the indirect method, based on the Kirchhoff’s law, for opaque objects, the summation of emissivity and reflectivity is one. So, one can measure the reflectance ρ of the sample first and then calculate the emissivity ε by using ε=1−ρ [5,6].

In the direct method, especially when the sample’s temperature is near room temperature, the measured signal of the sample might be affected by the background radiation, which can be expressed as Equation (1).

(1) $$L_{s}=\varepsilon L_{bb}+(1-\varepsilon )L_{ambient}+L_{0}$$

In this equation, $L_{s}$ and ε represent the radiance and emissivity of the sample to be measured, respectively, $(1-\varepsilon )L_{ambient}$ represents the radiance from the background environment $L_{ambient}$ reflected by the sample, and $L_{0}$ is the radiance directly from the background environment.

In recent years, some researchers have mounted an opto-mechanical unit in liquid nitrogen-cooled chambers, which can reduce stray radiation well, but the whole system is relatively complicated and cannot be used for on-site measurements [7,8,9]. Other researchers have adopted the multi- or (modified) two-temperature calibration methods [10,11,12]. For example, Del Campo et al. (2006) summarized the fact that emissivity could be calculated based on Equations (2) and (3) [10].

(2) $$S_{bb}({\lambda ,\;T}_{bb})=R(\lambda )[L({\lambda ,\;T}_{bb})+S_{0}(\lambda )]$$

(3) $$S_{s}\;({\lambda ,\;T}_{s})=R(\lambda )[\varepsilon_{s}({\lambda ,\;T}_{s})L({\lambda ,\;T}_{s})+(1-\varepsilon_{s}({\lambda ,\;T}_{s}))\varepsilon_{sur}L({\lambda ,\;T}_{sur})+S_{0}(\lambda )]\;$$

In these, $S_{bb}({\lambda ,\;T}_{bb})$ and $S_{s}({\lambda ,T}_{s})$ are the measured signals of the blackbody and the sample, respectively; $T_{s}$ is the temperature of the sample to be measured; $L({\lambda ,T}_{bb})$ is the spectral radiance calculated based on Planck’s law at temperature $T_{bb}$ and wavelength λ; R(λ) and $S_{0}(\lambda )$ are the response function of the measurement apparatus and the background radiation, respectively; $\varepsilon ({\lambda ,T}_{s})L({\lambda ,T}_{s})$ is the radiation emitted by the sample; $\varepsilon_{s}({\lambda ,T}_{s})$ is the emissivity of the sample to be measured; $\left(1-\varepsilon_{s}({\lambda ,T}_{s}))\varepsilon_{sur}L({\lambda ,T}_{sur}\right)$ represents the radiant signal from the surroundings reflected by the sample; $\varepsilon_{sur}$ and $T_{sur}$ are the emissivity and the temperature of the surroundings; and $L({\lambda ,T}_{s})$ and $L({\lambda ,T}_{sur})$ are the radiances at temperatures $T_{s}$ and $T_{sur}$, respectively, calculated based on Planck’s law [10].

In their study, the emissivity of the surroundings $\varepsilon_{sur}$ was assumed to be independent of temperature and wavelength, and the background radiation $S_{0}(\lambda )$ was considered to be independent of temperature. By separately measuring the blackbody at two different temperatures, $T_{bb}$ and ${T\prime }_{bb}$, following Equation (2), and combining these with Equation (3), the sample’s emissivity can be calculated accordingly [10].

However, since the background radiation is not stable, and there is also a zero-point deviation for the Fourier-transform Infrared Radiometer (FTIR) during measurements, the above assumption might induce some deviations in the measured emissivity [12]. To solve these problems, Zhang and Liu (2020) proposed a modified two-temperature calibration method. In their method, they used a main blackbody and one more assisted blackbody to eliminate the background radiation and the zero-point deviation of the FTIR [12]. However, two blackbodies were used in their facility, which made the system complex and high-cost.

To simplify the whole system, this study develops another modified two-temperature calibration method and facility for emissivity measurement by setting the two temperature points of the sample with a certain small interval. At these two temperatures, the background radiation and the emissivities of the sample are considered to remain the same (for samples with emissivities that are temperature insensitive, or only have gradual changes along with the changes in temperature). With this method, a blackbody is not needed in the system, and the facility can be built up in a simple and cost-effective way, but with a relatively high measurement accuracy. Details will be presented underneath.

## 2. The Principle of the Developed Modified Two-Temperature Calibration Method

This study proposes a modified two-temperature calibration method and facility for emissivity measurement. With this method, the radiant signals of the sample at different temperatures ($T_{1}$ and $T_{2}$) can be measured and expressed as Equations (4) and (5).

(4) $$L_{s}(T_{1})=\varepsilon (T_{1})\;L_{bb}(T_{1})+(1-\varepsilon (T_{1}))L_{ambient}(T_{a1})+L_{a1}$$

(5) $$L_{s}(T_{2})=\varepsilon (T_{2})\;L_{bb}(T_{2})+(1-\varepsilon (T_{2}))L_{ambient}(T_{a2})+L_{a2}\;$$

In these, $T_{1}$ and $T_{2}$ represent the two temperature points, $L_{s}(T_{i})$ and $L_{bb}(T_{i})$ represent the radiant signals from the sample and an ideal blackbody, respectively, $\varepsilon (T_{i})$ and $\left(1-\varepsilon (T_{i}))L_{ambient}(T_{ai}\right)$ represent the sample’s emissivity and the radiance of the background environment reflected by the sample at temperature $T_{i}$, respectively, $L_{ai}$ and $L_{ambient}(T_{ai})$ represent the stray radiance coming directly from the background environment and the incidents on the sample at $T_{i}$, i = 1, 2, respectively.

The principle of the developed modified two-temperature calibration method is that the two temperatures are set in a certain small interval, it only takes a short time to increase the temperature of the sample from $T_{1}$ to $T_{2}$; so it is assumed that the emissivities of the sample are the same at these two temperatures, and so are the radiant signals reflected by the sample and the stray radiations from the background environment, which can be expressed as Equations (6)–(8).

(6) $$\varepsilon (T_{2})\;\approx \;\varepsilon (T_{1})$$

(7) $$L_{ambient}(T_{a2})\;\approx \;L_{ambient}(T_{a1})\;$$

(8) $$L_{a2}\;{\approx L}_{a1}$$

Based on the above approximation, Equations (4) and (5) can be expressed as Equation (9):

(9) $$\varepsilon (T_{2})\;\approx \;\frac{L_{s}(T_{2})-L_{s}(T_{1})\;}{L_{bb}(T_{2})-L_{bb}(T_{1})}$$

According to the Stefan–Boltzmann law, the above equation can be further expressed as Equation (10):

(10) $$\begin{array}{c} \varepsilon (T_{2})\;\approx \;\frac{L_{s}(T_{2})-L_{s}(T_{1})\;}{L_{bb}(T_{2})-L_{bb}(T_{1})}=\frac{T_{s2-radiant}^{4}-T_{s1-radiant}^{4}\;}{T_{bb2}^{4}-T_{bb1}^{4}}\\ =\frac{T_{s2-radiant}^{4}-T_{s1-radiant}^{4}\;}{T_{s2-contact}^{4}-T_{s1-contact}^{4}} \end{array}$$

In this, $T_{s1-radiant}$ (unit: K) and $T_{s2-radiant}$(unit: K) represent the radiant temperatures of the sample at temperatures $T_{1}$ and $T_{2}$, respectively, and $T_{bb1}$ (unit: K) and $T_{bb2}$(unit: K) represent the temperatures of an ideal blackbody, which can be considered the same as the contact temperatures of the sample, $T_{s1-contact}$ (unit: K) and $T_{s2-contact}$(unit: K). Based on Equation (10), the temperatures $T_{s1-radiant}$ and $T_{s2-radiant}$ can be measured with an FTIR or a radiant thermometer, and the temperatures $T_{s1-contact}$ and $T_{s2-contact}$ can be measured with a contact thermometer, like a Platinum resistance thermometer.

As mentioned before, we made the approximation by controlling the temperatures $T_{1}$ and $T_{2}$ in a certain small interval. So, how one sets the temperature interval $∆T\;(∆T=T_{s2-contact}-T_{s1-contact}$) is important. Equation (11) shows the deduction from Equation (10).

(11) $$\begin{array}{c} \;\;\;\;\;\;\;\;\;\;\;\;\;\;\;∆T=T_{s2-contact}-T_{s1-contact}\\ \approx \;\frac{{(T}_{s2-radiant}^{2}+T_{s1-radiant}^{2})\;\cdot \;\left(T_{s2-radiant}+T_{s1-radiant}\right)\;\cdot \;(T_{s2-radiant}-T_{s1-radiant})}{\varepsilon \;\cdot \;\left(T_{s2-contact}^{2}+T_{s1-contact}^{2}\right)\;\cdot \;(T_{s2-contact}+T_{s1-contact})}\\ \leq \frac{\;(T_{s2-radiant}-T_{s1-radiant})}{\varepsilon } \end{array}$$

From Equation (11), to calculate the ∆T, an estimated emissivity value ε can be used based on some priori knowledge; for the ${∆T}_{radiant}={(T}_{s2-radiant}-T_{s1-radiant}$), it is mainly determined by the measurement uncertainty of the radiant thermometer. Although we want to control the ∆T (namely ${∆T}_{radiant}$) in a small interval, the gap between $T_{s2-radiant}$ and $T_{s1-radiant}$ should be large enough to make sure these two temperatures can be distinguished as two temperatures, while not within the uncertainty zone of each temperature. By referring to the decision rules for proving conformity or nonconformity with the specifications defined in the international standard ISO 14253 and also the tolerance between the two measurement results [13], in the developed method, it is recommended that the interval between these two temperatures should be no smaller than twice the expanded uncertainty of the radiant thermometer (*k* = 2) [13]. If the emissivity at temperature $T_{2}$ needs to be measured, we can make a measurement at the temperature points $T_{2}$ and $T_{2}-∆T$. For example, if the expanded measurement uncertainties *U* of the radiant thermometer at temperatures $T_{s1-radiant}$ and $T_{s2-radiant}$ are both 0.2 °C (*k* = 2), then the suggested ${∆T}_{radiant}$ is ${∆T}_{radiant}\geq 2U=0.4{}^{\circ}C$. So, for a low emissivity sample (like ε = 0.100), the ∆T should be no smaller than 4.0 °C based on Equation (11); for a high emissivity sample (like ε = 0.900), the ∆T should be no smaller than 0.5 °C. If the priori knowledge about the emissivity value of the sample is unknown, we can perform the following: at first, set the ∆T  to a certain value like 10.0 °C; make measurements and calculate a preliminary emissivity ε; then use this ε as the estimated emissivity to recalculate the ∆T; re-measure the emissivity accordingly; and perform an iteration if needed.

Based on Equation (11), the temperature interval ∆T is inversely proportional to the emissivity of the sample. So, for a low emissivity sample, the ∆T will be large; while for a high emissivity sample, the ∆T will be small. However, when doing the uncertainty analysis based on Equation (10), it can be found the small ∆T might cause a relatively large measurement uncertainty. Since, for a high emissivity sample, the background radiation has less influence on its emissivity measurement, the enlarged temperature interval ∆T will not have an obvious influence on the emissivity measurement, but can improve the measurement uncertainty to a certain extent. Hence, in the developed method, we used the ∆T calculated at low emissivity (like ε = 0.100) as the ∆T for the samples whose emissivities were larger than 0.100, so Equation (11) can be expressed as Equation (12).

(12) $$∆T\;\geq \;\frac{2U_{2}}{\varepsilon }=\frac{2U_{2}}{0.100}$$

In this, $U_{2}$ is the expanded uncertainty of the radiant thermometer (*k* = 2) at temperature $T_{2}$, and ε is the lower limit of our measurement range, ε = 0.100. Furthermore, we also gave some margin for the ∆T just in case there is some deviation of the estimated emissivity. We did not evaluate and compare those samples whose emissivities are smaller than 0.100 (because of the relatively large uncertainty in a near-room temperature condition), so it is recommended that this developed method is used for emissivity measurements in the range of (0.100~0.999).

## 3. Experiments and Uncertainty Estimation

### 3.1. Experimental Facility and Results

**Figure 1:**
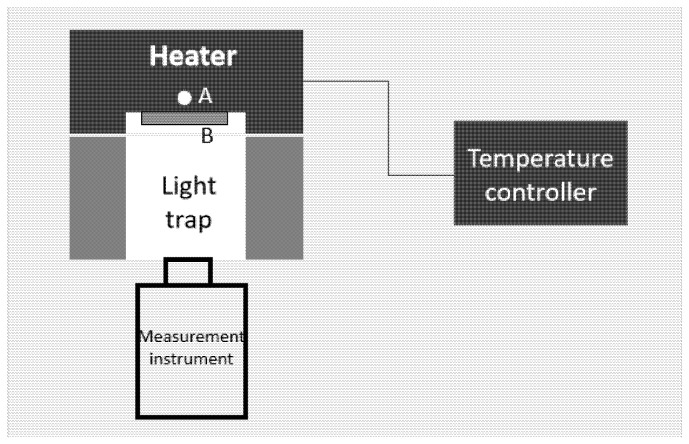
Schematic of the developed experimental facility (Top view). The white circle “A” indicates the hole for the contact thermometer, and the yellow rectangle “B” represents the sample to be measured, which is installed at the front surface of the heater.

**Figure 2:**
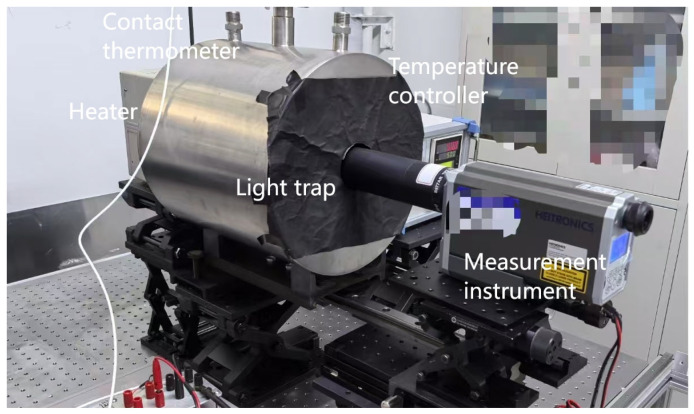
Photograph of the developed experimental facility.

Based on the modified two-temperature calibration method proposed above, we constructed the experimental facility as the schematic (top view) shown in Figure 1. The heater was used to heat the sample to be measured. The white circle “A” indicates the hole for the contact thermometer (a Platinum resistance thermometer in the developed facility) with the contact point at the geometric center of the heater; the yellow rectangle “B” represents the sample to be measured, which is installed at the front surface of the heater; and the temperature controller was used to control the temperature of the heater. A light trap was put between the heater and the measurement instrument. The opening diameter was the same as that of the heater, so it could fit well with the heater to reduce the air convection on the sample surface during the measurement. The inner wall of the light trap was painted with a high emissivity coating to absorb stray radiation from the sample or the background environment. It was moved away from the heater during the heating period, and pushed back to the heater during the measurement period. The measurement should be finished in a short time (in tens of seconds) to avoid radiation changes in the light trap. The measurement instrument can be a radiant thermometer or an FTIR. A photograph of the developed facility is shown in Figure 2.

In the developed facility, the measurement instrument is an infrared radiant thermometer. Its wavelength range is (8~14) μm, the working distance is 380 mm, the optical field of view is 6.8 mm, and the temperature range is (−150~1000) °C. Since the deduction of Equation (10) is based on the Stefan–Boltzmann law in the full wavelength range condition, although the radiant thermometer is in a specific narrow spectral band, it was calibrated with a standard blackbody in small temperature steps. So, if the sample to be measured is a gray body (similar spectral distribution as a standard blackbody), the temperature measured with this thermometer should have negligible deviation when comparing it to the value measured by a full-band thermometer. As a result, in the developed experiments, we used gray bodies as the samples to be measured and marked the results in (8~14) μm. Moreover, we have also compared our results with those measured with other methods.

Regarding the contact thermometer, since the measurement uncertainty of the surface probe thermometer (*U* = 0.9 K at 100 °C, *k* = 2) is much larger than that of the Platinum resistance thermometer (*U* = 0.010 K at 100 °C, *k* = 2), and the contact of the surface probe might destroy the surface (especially the coating at the surface) of the sample, we used a Platinum resistance thermometer instead of a surface probe thermometer.

**Figure 3:**
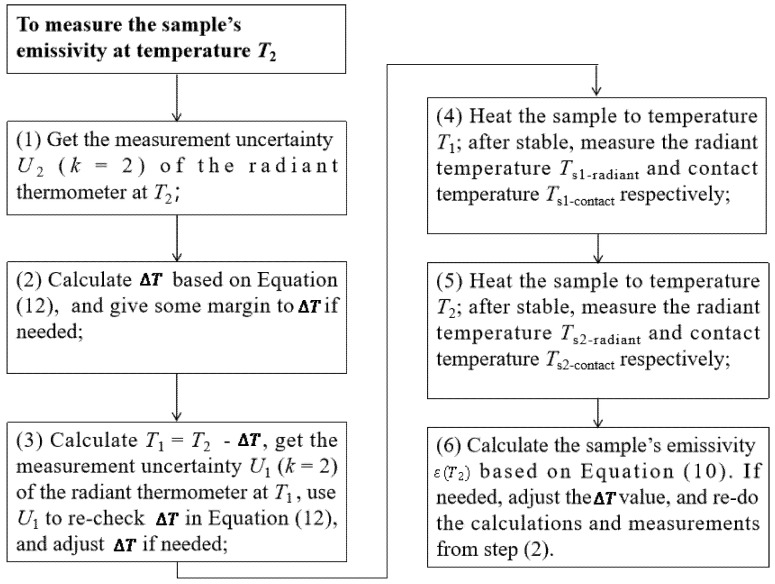
Emissivity measurement procedure based on the developed method.

The whole procedure can be expressed through the flowchart in Figure 3. Based on the above steps, we measured three samples (samples 1–3) at different temperatures (60 °C and 85 °C). Their estimated emissivities are 0.100, 0.500, and 0.900, respectively. For the radiant thermometer, the measurement uncertainties are 0.2 °C (*k* = 2) at 60 °C and 0.3 °C (*k* = 2) at 85 °C, respectively, so the temperature interval ∆T can be set to 4.0 °C (60 °C) and 6.0 °C (85 °C) for both samples. After taking under consideration the fact that the emissivity estimation might have some deviation, we gave some margin for each ∆T, set as 10.0 °C (60 °C) and 15.0 °C (85 °C) for both samples. The detailed information for each sample is listed in Table 1 [14].

Since there are temperature drops from the measurement point to the surface of the heater (from hole A to the front surface of the heater in Figure 1, with a thickness of 10 mm, and comprising copper), and also from the heater’s surface to the sample’s surface (from the front surface of the heater to the front surface of B in Figure 1), for samples 2 and 3, the gray and black coatings may also cause a temperature drop; so, we calculated the temperature drops based on the heat transfer equation as expressed in Equation (13) for each sample at each temperature point [15,16]. And then we used these values to correct the temperature measured by the contact thermometer, and calculated the emissivity based on Equation (10).

(13) $${∆T}_{drop}=\varepsilon \sigma \left(T_{s-contact}^{4}-T_{amb}^{4}\right)\;\cdot d/ĸ$$

In this, ${∆T}_{drop}$ represents the temperature drop; ε represents the emissivity of the heater or sample; σ represents the Stefan–Boltzmann constant, $\sigma =5.670\times 10^{-8}\;W/(m^{2}\times K^{4})$; $T_{s-contact}$ and $T_{amb}$ represent the temperatures of the sample and the background environment; *d* is the thickness of the heater or the sample; and ĸ is the thermal conductivity of the heater material or sample. In the experiments, a light trap was used which can control the stray radiation in a relatively stable condition and reduce the air convection on the sample surface as well.

As listed in Table 2, the experimental results show that, for sample 1, the measured emissivities are 0.110 at 60 °C and 0.114 at 85 °C, respectively; for sample 2, the measured emissivities are 0.529 at 60 °C and 0.527 at 85 °C, respectively; and for sample 3, the measured emissivities are 0.932 at 60 °C and 0.933 at 85 °C, respectively.

### 3.2. Uncertainty Estimation

The measurement uncertainty with the developed method was estimated as shown in Table 3 [17,18,19]. The repeatabilities were calculated with ten repeated measurements in a short time using the Bessel’s formula. The reproducibilities were calculated with at least four different days’ results using the range method. By calculating the partial derivative of $\varepsilon (T_{2})$ with respect to $T_{s1-contact}$, $T_{s2-contact}$, $T_{s1-radiant}$, and $T_{s2-radiant}$ separately based on Equation (10), the sensitivity coefficient for each parameter related to the emissivity can be obtained, and then the uncertainty budget caused by each parameter can be calculated accordingly. The stabilities were calculated with 20 min data using the range method. The impact of the uncertainties of the temperature drops on the emissivity measurement were evaluated based on Equation (13). Moreover, we also measured the emissivities in the left, right, top, and bottom areas of each sample, and calculated their absolute values by subtracting the emissivity in the center area from the emissivity in each area. Uniformity was calculated with the maximum value among these absolute values. Although a light trap was used to control the stray radiation and reduce the air convection on the sample surface, the uncertainty budget was estimated for each sample.

After calculation, the combined relative expanded uncertainties are 9.6% (*k* = 2) at 60 °C and 8.8% (*k* = 2) at 85 °C for sample 1, 4.0% (*k* = 2) at 60 °C and 5.8% (*k* = 2) at 85 °C for sample 2, and 1.5% (*k* = 2) at 60 °C and 1.2% (*k* = 2) at 85 °C for sample 3. A previous study constructed a vacuum and an FTIR measurement system with a liquid-nitrogen cooled detector, and reported that at 100 °C the combined standard relative uncertainties were less than 0.50% (*k* = 1) when emissivity was larger than 0.9, and less than 3% (*k* = 1) when emissivity was approximately 0.2 [17]. The developed facility was neither in a vacuum condition, nor with liquid-nitrogen cooled system, and the experiments were implemented at even lower temperatures (lower than 100 °C); but similar levels of measurement uncertainties were obtained, suggesting the effectiveness of the developed method.

## 4. Comparisons and Discussions

To evaluate the effectiveness of the developed method, we also measured the same samples with the indirect method and the conventional two-temperature calibration method. In the indirect method, an FTIR containing a reflective integrating sphere accessory was used in a 0/d measurement condition, so the hemispherical emissivity could be obtained with this system. The samples were measured at room temperature (24 °C), and the emissivities were calculated in the (8~14) μm wavelength range. In the conventional two-temperature calibration method, as described in Section 1 [10], a blackbody was used and set at two temperatures (*T*_bb_ = 30 °C and *T’*_bb_ = 60 °C for sample emissivity measurements at 60 °C, and *T*_bb_ = 30 °C and *T’*_bb_ = 85 °C for sample emissivity measurements at 85 °C). The radiant and contact temperatures were measured with the same instruments used in the developed method, and the emissivities were calculated based on Equations (2) and (3).

Since the results measured with the conventional two-temperature calibration method and the developed method are normal emissivities, we also converted the normal emissivities into hemispherical emissivities by referring to a previous study [20]. The experimental results are listed in Table 4.

To further analyze the consistency of the experimental results measured with the above three methods, we also calculated the normalized error *E*n values with the hemispherical emissivities of the three methods for each sample in (8~14) μm. Based on the definition of the *E*n value, consistency is determined: if it is smaller than one, it suggests consistency in the emissivity values measured with these methods. The results are shown in Table 5.

From Table 4 and Table 5, when comparing the hemispherical emissivities measured with the three methods, all the *E*n values are smaller than one, suggesting the consistency of the three methods. However, for sample 1 (relatively low emissivity sample), the emissivity values measured with the conventional two-temperature calibration method are the largest (${∆\varepsilon }_{max}=0.064$, *E*n = 0.89 and 0.71). This might be explained with the following reasons. According to the principle of the conventional two-temperature calibration method as described in Section 1, the background radiation was considered to be constant in this method. However, since a blackbody was heated at different temperatures in this method, this might change the background radiation and also increase the measurement uncertainties [12]. During experiment, when the temperature of the blackbody was increasing from *T*_bb_ to *T*’_bb_, the increased background radiation might be processed as the radiation from the sample, thus inducing the larger emissivity values. Especially for the low emissivity sample (like sample 1), and in the near-room temperature condition (at 60 °C), the background radiation has an even larger influence on the emissivity measurement than the sample self. Any shift in background radiation will cause obvious changes in emissivity values. This might be why there is a 0.010 (0.173 − 0.163 = 0.010) shift in the emissivity values between 60 °C and 85 °C. The above results suggest that the conventional two-temperature calibration method might be used for high-temperature emissivity measurement, since the influence of background is relatively low and might be considered constant in this condition; but this is not true for the near-room temperature emissivity measurement. Additionally, the above results also suggest the effectiveness of the developed method.

## 5. Novel Points and Limitations of This Study

### 5.1. Novel Points of This Study

Different from the conventional two-temperature calibration method, in the developed method, the two temperatures ($T_{1}$ and $T_{2}$) are controlled in a certain small interval based on the developed calculation method, it only takes a short time (around ten minutes in this study) to heat the sample from $T_{1}$ to $T_{2}$, so the stray radiation from the background environment and the temperature drops of the sample surface will have no obvious change. And the emissivity at $T_{2}$ is calculated based on the radiant variations between these two temperatures, so we do not need to measure the exact values of the stray radiation from the background environment, or the temperatures of the blackbody and so on. Moreover, the uncertainty budget and the stray radiation can be reduced because there is no standard blackbody in the developed system. As a result, the measurement uncertainty of the whole system is relatively low in the developed system. The whole facility is simplified and portable. Therefore, the developed method and facility might be suitable not only for laboratory use, but also for on-site measurement, especially in the condition where the background environment experiences changes. Additionally, the developed method and facility can also be used for a directional emissivity measurement.

### 5.2. Limitations of This Study and Future Directions

Although the two temperatures were controlled in a small interval and a light trap was used to reduce the stray radiation and air convection on the sample surface, we made an approximation that the radiant signals reflected by the sample and the stray radiations from the background environment remain the same at these two temperatures. We also made an approximation that the emissivities of the sample at $T_{1}$ and $T_{2}$ are the same, so this method might be suitable for those samples whose emissivities are temperature insensitive, but might not for certain other materials (like phase-change materials).

Moreover, as explained in Section 3.1, the Stefan–Boltzmann law is applied in a full wavelength range, but the wavelength range of our radiant thermometer is (8~14) μm. To solve this problem, we used a standard body to calibrate the thermometer, adopted gray body samples to perform the experiments, and also reported the emissivities in (8~14) μm wavelength condition.

Although there are several limitations in the developed method and current facility, the developed method is suggested to be effective. For the next steps, we may perform the following: (1) use an FTIR which works in a wider wavelength range (to replace the radiant thermometer) to re-evaluate this method; (2) study a specific formula to solve the narrow spectral window problem of the radiometer when using the Stefan–Boltzmann law [21]; and (3) improve the developed facility for both normal/directional and hemispherical emissivity measurement.

## 6. Conclusions

This study proposes a modified two-temperature calibration method and facility for emissivity measurement, in which, (1) the two temperature points are set in a certain small interval by calculating with the uncertainty of the measurement instrument (like an infrared radiant thermometer) and the estimated emissivity of the sample with the developed method; (2) based on the indication of the approximation that the emissivities of the sample and the background radiations remain the same at these two temperatures, the emissivities can be calculated by using the radiant and contact signals at these two temperatures; and (3) a blackbody is not needed in the developed facility, so the measurement uncertainty can be reduced accordingly and the whole system is simplified and portable. With this method, the influences (like background radiation, measurement uncertainties from the blackbody or from the sample surface temperature, and so on) on emissivity measurement can be reduced or eliminated. Three samples with emissivities around 0.100, 0.500, and 0.900 were measured with the developed method in (8~14) μm. And the relative expanded uncertainties were 9.6%, 4.0%, and 1.5% at 60 °C (*k* = 2), respectively, and 8.8%, 5.8%, and 1.2% at 85 °C (*k* = 2), respectively. The experimental results were compared with those measured with the indirect method and the conventional two-temperature calibration method, and indicate the effectiveness of the developed method. The developed method and facility might be suitable for both laboratory and on-site measurements, and for samples whose emissivities are temperature insensitive.

## Figures and Tables

**Table 1:** Information of samples 1–3 in the developed experiments.

Parameters	Sample 1	Sample 2	Sample 3
Material	Aluminum plate	Aluminum plate with gray coating	Stainless steel with black coating
Thickness (mm)	0.5	2.2 (Aluminum) 0.1 (self-made gray coating)	3 (Stainless steel) 0.1 (black coating)
Estimated emissivity (ε)	0.100	0.500	0.900
∆T at 60 °C (°C)	10.0	10.0	10.0
∆T at 85 °C (°C)	15.0	15.0	15.0
Thermal conductivity (W·m^−1^·K^−1^)	205	205 (Aluminum) 20 (self-made gray coating)	15 (Stainless steel) 0.2 (black coating)

**Table 2:** Measured emissivities of samples 1–3 with the developed method in (8~14) μm.

**Samples**	Set Value (°C)		Contact Thermometer $T_{si-contact}$ (°C)	Radiant $\mathrm{Thermometer}\;T_{si-radiant}$ (°C)	$\mathrm{Emissivity}\;\mathrm{in}\;(8\sim 14)\;\mu m\;\mathrm{at}\;T_{2}$ Calculated Based on the Developed Method (ε)
Sample 1	$T_{1}$	50	51.77	28.7	0.110
	$T_{2}$	60	62.50	30.3	
	$T_{1}$	70	73.28	31.3	0.114
	$T_{2}$	85	89.30	34.2	
Sample 2	$T_{1}$	50	51.53	40.2	0.529
	$T_{2}$	60	62.28	46.6	
	$T_{1}$	70	73.08	52.9	0.527
	$T_{2}$	85	89.32	63.3	
Sample 3	$T_{1}$	50	51.75	50.1	0.932
	$T_{2}$	60	62.48	60.3	
	$T_{1}$	70	73.28	70.5	0.933
	$T_{2}$	85	89.36	85.9	

**Table 3:** Uncertainty estimations of emissivity measurements for samples 1–3.

Uncertainty Component	Sample 1		Sample 2		Sample 3	
	60 ℃	85 ℃	60 ℃	85 ℃	60 ℃	85 ℃
Repeatability (%)	1.08	0.60	0.20	0.33	0.19	0.11
Reproducibility (%)	2.62	2.17	0.74	0.86	0.59	0.34
$\mathrm{Uncertainty}\;\mathrm{of}\;T_{s1-contact}$ measurement (%)	0.00003	0.00007	0.00014	0.00030	0.00026	0.00054
$\mathrm{Uncertainty}\;\mathrm{of}\;T_{s2-contact}$ measurement (%)	0.00003	0.00008	0.00015	0.00035	0.00027	0.00062
$\mathrm{Uncertainty}\;\mathrm{of}\;T_{s1-radiant}$ measurement (%)	0.0024	0.0059	0.0025	0.0072	0.0027	0.0085
$\mathrm{Uncertainty}\;\mathrm{of}\;T_{s2-radiant}$ measurement (%)	0.0024	0.0061	0.0026	0.0079	0.0029	0.0097
$\mathrm{Stability}\;\mathrm{at}\;T_{1}$ (%)	0.0002	0.0003	0.0015	0.0044	0.0031	0.0069
$\mathrm{Stability}\;\mathrm{at}\;T_{2}$ (%)	0.0002	0.0004	0.0015	0.0048	0.0033	0.0079
$\mathrm{Temperature}\;\mathrm{drop}\;\mathrm{at}\;T_{1}$ (%)	N.S. *	N.S. *	0.00002	0.00002	0.00294	0.00342
$\mathrm{Temperature}\;\mathrm{drop}\;\mathrm{at}\;T_{2}$ (%)	N.S. *	N.S. *	0.00002	0.00002	0.00447	0.00529
Uniformity (%)	3.64	3.54	1.70	2.66	0.32	0.43
Stray radiation and air convection on the sample surface (%)	1.0	1.0	0.5	0.5	0.1	0.1
Combined uncertainty (*u*_rel_, %)	4.8	4.4	2.0	2.9	0.71	0.57
Combined relative expanded uncertainty (*U*_rel_, %, *k* = 2)	9.6	8.8	4.0	5.8	1.5	1.2

N.S. *: negligibly small.

**Table 4:** Emissivity values of samples 1–3 measured with three methods in (8~14) μm.

Samples	The Indirect Method	The Conventional Two-Temperature Calibration Method		The Developed Method	
	Measured Hemispherical Emissivity (ε)	Measured Normal Emissivity (ε)	Estimated Hemispherical Emissivity (ε)	Measured Normal Emissivity (ε)	Estimated Hemispherical Emissivity (ε)
Sample 1	0.138 (24 °C)	0.173 (60 °C) 0.163 (85 °C)	0.192 (60 °C) 0.182 (85 °C)	0.110 (60 °C) 0.114 (85 °C	0.128 (60 °C) 0.132 (85 °C)
Sample 2	0.529 (24 °C)	0.552 (60 °C) 0.537 (85 °C)	0.540 (60 °C) 0.527 (85 °C)	0.529 (60 °C) 0.527 (85 °C)	0.520 (60 °C) 0.519 (85 °C)
Sample 3	0.916 (24 °C)	0.941 (60 °C) 0.943 (85 °C)	0.911 (60 °C) 0.914 (85 °C)	0.932 (60 °C) 0.933 (85 °C)	0.898 (60 °C) 0.899 (85 °C)

**Table 5:** *E*n values of samples 1–3 measured with three methods in (8~14) μm.

Samples	*E*_n_ Values of the Indirect Method	*E*_n_ Values of the Conventional Two-Temperature Calibration Method	*E*_n_ Values of the Developed Method
Sample 1	0.33 (24 °C)	0.89 (60 °C)	0.56 (60 °C)
	0.29 (24 °C)	0.71 (85 °C)	0.42 (85 °C)
Sample 2	0.01 (24 °C)	0.22 (60 °C)	0.20 (60 °C)
	0.08 (24 °C)	0.04 (85 °C)	0.12 (85 °C)
Sample 3	0.17 (24 °C)	0.06 (60 °C)	0.23 (60 °C)
	0.15 (24 °C)	0.10 (85 °C)	0.23 (85 °C)

## Data Availability

The original contributions presented in this study are included in the article. Further inquiries can be directed to the corresponding authors.
